# Investigation of an outbreak of COVID-19 among U.S. military personnel and beneficiaries stationed in the Republic of Korea, June-July 2021

**DOI:** 10.1371/journal.pgph.0000236

**Published:** 2022-05-27

**Authors:** Shilpa Hakre, Evelyn Y. Lam, Irina Maljkovic-Berry, Jun Hang, Luis A. Pow Sang, Elizabeth J. Bianchi, Christian Fung, Jay Gandhi, Marcus Chibucos, Matthew A. Conte, Adam R. Pollio, Christine A. Mariskanish, Luke A. Mansfield, Kayvon Modjarrad, Heather L. Friberg-Robertson, Grace M. Lidl, Paul T. Scott, Douglas A. Lougee

**Affiliations:** 1 Walter Reed Army Institute of Research, Emerging Infectious Diseases Branch, Silver Spring, Maryland, United States of America; 2 Henry M. Jackson Foundation for the Advancement of Military Medicine, Inc., Bethesda, Maryland, United States of America; 3 United States Forces Korea, J3 –Directorate of Operations, Camp Humphreys, Pyeongtaek, South Korea; 4 Walter Reed Army Institute of Research, Viral Diseases Branch, Silver Spring, Maryland, United States of America; 5 Cherokee Nation Technology Solutions, Tulsa, Oklahoma, United States of America; 6 United States Forces Korea, Brian D. Allgood Army Community Hospital, Camp Humphreys, Pyeongtaek, South Korea; Kuwait University, KUWAIT

## Abstract

On 28 May 2021, leisure travel restrictions in place to control coronavirus disease 2019 (COVID-19) were eased among vaccinated U.S. military personnel and beneficiaries stationed in South Korea (USFK) allowing access to bars and clubs which were off limits. We describe results from an investigation of the largest severe acute respiratory syndrome coronavirus 2 (SARS-CoV-2) outbreak as of November 2021 among USFK personnel following this change in policy. Data such as SARS-CoV-2 real-time polymerase chain reaction (RT-PCR) test results, demographic characteristics, symptom and vaccination histories, and genome sequences were analyzed. Of a total 207 new cases of COVID-19 diagnosed among USFK members from 15 June to 27 July 2021, 113 (57%) eligible cases were fully vaccinated, of whom 86 (76%) were symptomatic. RT-PCR cycling threshold values were similar among vaccinated and unvaccinated members. Whole genomic sequencing of 54 outbreak samples indicated all infections were due to the Delta variant. Phylogenetic analysis revealed two sources of SARS-CoV-2 accounted for 41% of infections among vaccinated and unvaccinated members. Vaccinated personnel were not at risk of severe illness; however, 86% experienced symptoms following infection. There were no hospitalizations among COVID-19 cases, most of whom were young military service members. Rescinded restrictions were reinstated to control the outbreak. Masking was mandated among all personnel predating U.S. national recommendations for indoor masking in high COVID-19 transmission areas. Increased vaccination with continued vigilance and extension of COVID-19 mitigation measures are warranted to contain the spread of SARS-CoV-2 variants of concern.

## Introduction

Countries have implemented several measures to contain and control outbreaks since the emergence of Severe Acute Respiratory Syndrome Coronavirus 2 (SARS-CoV-2) in December 2019 and proclamation by the World Health Organization (WHO) on 30 January, 2020 that Coronavirus disease 2019 (COVID-19) was a Public Health Emergency of International Concern [1]. Due to lessons learned from managing the Middle East respiratory syndrome (MERS) outbreak in 2015, the Republic of Korea (ROK) was well prepared to control the coronavirus disease 2019 (COVID-19) outbreak nationally, without the stringent national lockdowns other countries implemented, using a strategy of “test, trace, isolate” following identification of the first case on January 20, 2020 [2, 3]. Simultaneously, the United States Forces Korea (USFK) implemented multiple robust COVID-19 mitigation measures involving post-arrival screen testing, post-arrival 14-day self-quarantine, isolation of cases in designated quarters, test based clearance from isolation, surveillance testing, restrictions on high risk activities and leisure travel restrictions, as well as masking, physical distancing, and hand hygiene [4]. In addition, since the U.S. Food and Drug Administration (FDA) granted emergency use authorization (EUA) for COVID-19 vaccines, inoculations were administered, as vaccine supplies allowed and in accordance with DoD policy, to eligible USFK personnel and beneficiaries who opted in. The ROK enacted pre-arrival screen testing policy in January 2021. As a result of implementation of both countries’ policies, USFK experienced a minimal number of COVID-19 cases during the first year of the pandemic [4].

Following lifting of a masking mandate among vaccinated personnel on 14 May 2021, USFK also eased most off-installation leisure travel-related COVID-19 restrictions in this vaccinated population, effective 28 May, permitting access to local bars and clubs in the greater Seoul metropolitan area [4]. Shortly thereafter, beginning 15 June, Army Public Health began receiving an increased number of COVID-19 positive reports among military personnel from Camp Humphreys, Pyeongtaek. We describe the epidemiology of the COVID-19 outbreak and public health response for containment of the virus.

## Methods

The United States Forces Korea, headquartered in Pyeongtaek, is staffed by more than 28,000 personnel from all branches of the United States military and Korean Augmentation to the United States Army program (KATUSA); the KATUSA program was initiated during the Korean war to address personnel shortages in the U.S. Army and includes enlisted Soldiers and non-commissioned officers. Personnel are stationed at 5 installations, the largest of which is staffed at over 20,000 troops. The Command Surgeon and staff develop and/or implement policies and programs to protect the force from disease and injury. Vaccinations were administered on a tiered scheduled in accordance with DoD policy to personnel who volunteered for the program. The Moderna vaccine was available at the start of the COVAX program in December 2020. Janssen vaccines became available following the U.S. Food and Drug Administration (FDA) Emergency Use Authorization (EUA) on February 27. The Pfizer vaccine was received only once during the time covered in this report, following EUA-approval for adolescents aged 12 to 17. As a result, vaccine coverage among USFK personnel in the month preceding the outbreak had reached almost 70%.

On 15 June 2021, the first local USFK case was reported at Camp Humphreys, Pyeongtaek after restrictions were lifted in May. Shortly thereafter on 17 June, 3 more local cases, all in the same battalion as the first cases, were reported marking the beginning of a cluster spread. Simultaneously, on 18 June at Camp Casey, Dongducheon, a second local case was reported followed by 2 more cases in the same unit, signaling the beginning of another cluster spread. Camp Casey is approximately 106 kilometers north of Camp Humphreys with the city of Seoul located between the two installations. During the early summer months of May and June, Seoul was the epicenter of rising cases of the fourth wave of COVID-19 in the Republic of Korea [5]. Exposure histories of USFK cases in June and early July indicated acquisition of SARS-CoV-2 was attributable to leisure activities in Seoul. Effective 9 July, shelter in place orders were issued reinstating travel restrictions to Seoul among all USFK-affiliated personnel [4]. The national incidence rate per 100,000 population had increased by 22 percent from the end of April (235.32) to July 27 (370.00) with a 67 percent increase in daily cases (627 to 1,896) during this time period [6, 7].

All positive COVID-19 test results were sent to USFK Public Health authorities for investigation of cases to elicit information regarding demographics, symptom onset, vaccination status, SARS-CoV-2 exposure history, and contact tracing. Interviews conducted elicited history of activities undertaken on- and off-installation locations in the 48–72 hours prior to onset of symptoms or, if asymptomatic, in the 4–7 days prior to confirmation of the first positive test result. Outbreak-related local cases were defined as any USFK/USFK-related individual with a positive reverse transcriptase polymerase chain reaction (RT-PCR) test result on/after 15 June who was not identified from travel-related testing and/or was epidemiologically linked to a confirmed case. All cases were isolated immediately in designated areas and all personnel working in the same unit as cases were mass tested with rapid antigen tests. Close/primary contacts were tested and required to quarantine for 7–14 days, depending on vaccination status and assessment of risk, and to test out negative: for vaccinated contacts, quarantining for 7 days RT-PCR negative on day 5 and antigen negative on day 7, for unvaccinated contacts, quarantining for 14 days, RT-PCR negative on day 12. Secondary contacts had to test negative (RT-PCR on day 1 and antigen on day 3) before resuming activities.

Confirmation of COVID-19 infection was by SARS-CoV-2 RT-PCR diagnostic testing (Cepheid GenXpert System/ BioFire System/ Hologic Panther platform assays) of nasopharyngeal (NP) specimens. Residual diagnostic specimens with a cycling threshold (Ct, inverse estimation of viral load) value below 31 were shipped to Walter Reed Army Institute of Research (WRAIR, Silver Spring, Maryland) for whole genome sequencing (WGS) and phylogenetic analysis. Ct values were unavailable for samples tested by Hologic Panther, BioFire or Korean private laboratories. Nucleic acids were extracted using MagMAX™ Pathogen RNA/DNA Kit (Life Technologies Corporation, Carlsbad, CA) and subjected to reverse transcription and PCR amplification using Fluidigm Juno System (Fluidigm, San Francisco, California) and next-generation sequencing using the Illumina MiSeq System reagents (Illumina, Inc., San Diego, California) [8]. Genomes were assembled using an ngs_mapper reference mapping pipeline (GitHub—VDBWRAIR/ngs_mapper: Genome Mapping Pipeline) with Pilon for indel detection, with a subsequent manual quality check. All genomes with 80% or greater full genome coverage were used in subsequent phylogenetic analyses. Each SARS-CoV-2 genome was matched to the top 20 most similar sequences in GISAID using BLAST. USFK genomes were aligned to a SARS-CoV-2 reference dataset from NextSTrain and to the top-matching BLAST genomes using MAFFT [9]. Sites that may interfere with accurate with phylogenetic inference were masked according to De Maio et al [10]. A maximum likelihood tree was inferred using GTR+gamma model of substitution using FastTree, with source code modified to display true branch lengths, and node support values were calculated using aLRT. Sustained spread was defined as three or more genomes within a monophyletic clade. Deidentified demographic characteristics, symptom, exposure and vaccination history, and positive screening results were described overall and by vaccination status. Diagnosis of COVID-19 14 days or more after completion of an FDA-approved vaccine series was defined as a breakthrough infection [11]. Accordingly anyone who did not receive a second dose in a two-dose series or anyone who received a single dose vaccine or a second dose within 14 days were considered partially vaccinated. Characteristics were compared statistically for differences by vaccination status using Chi-square for categorical variables and Kruskal-Wallis test for continuous variables. All analysis was performed with SAS version 9.4 (Cary, North Carolina) using anonymized data.

The WRAIR Human Subjects Protection Branch approved this project (WRAIR no. 2755) as a public health activity and not research. This work therefore was considered in support of public health surveillance not requiring informed consent.

## Results

**Fig 1:**
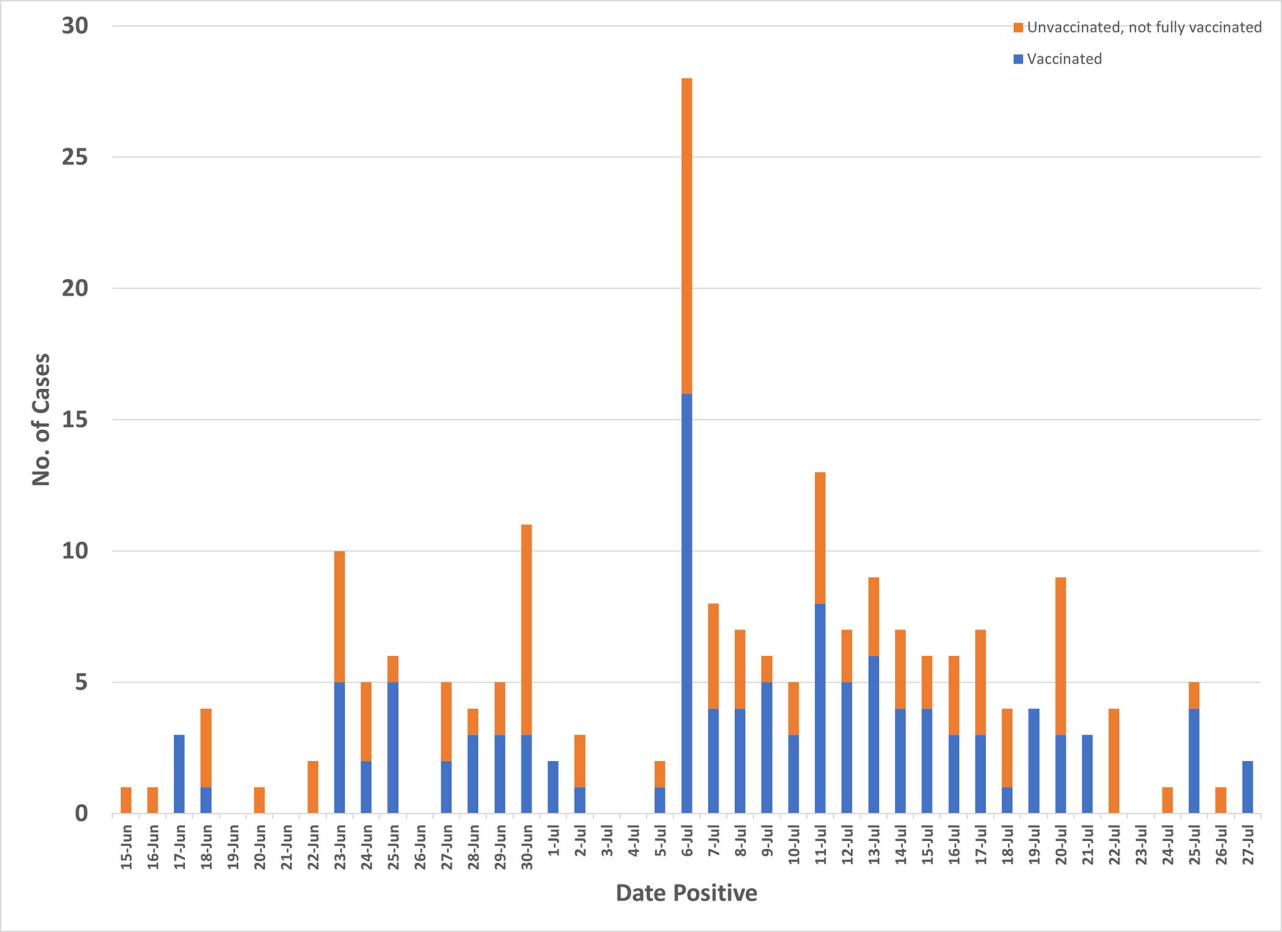
SARS-CoV-2 infections, by date of positivity and vaccination status, USFK, 15 June– 27 July, 2021.

A total 207 COVID-19 locally transmitted cases were identified in the outbreak among USFK/affiliated personnel and beneficiaries from 15 June to 27 July 2021 (Fig 1). Most outbreak cases were male (164, 79%), service members (169, 82%), of 20–29 years of age (55%, mean 26.7, range, 1.0–62.0), breakthrough infections (113/199, 57%), and identified from contact tracing (108, 52%) (Table 1). Eight cases were <12 years of age and ineligible for vaccination whereas 10 cases were vaccinated partially. Among 156 (75%) who reported symptoms of COVID-19, most reported cough (78, 50%), headache (64, 41%), congestion (64, 41%), and fever (52, 33%). No one was hospitalized and there were no reports of long-term COVID-19. Six military personnel had tested positive for COVID-19 previously (median interval between current and prior positive tests 187.5 days, range 124.0–293.0 days); two were vaccinated, two were vaccinated partially, and two were unvaccinated. Among cases with available RT-PCR results (n = 161), 137 (85%) specimens had Ct values below 31.0 (mean 22.1, range 10–44). Most cases (125, 60%) lived in shared housing on the installation whereas 16% (n = 33) lived elsewhere; housing information was unavailable for 49 cases (24%).

**Table 1:** Characteristics of 207 outbreak-related cases, overall, and by vaccination status, United States Forces Korea, June 15-July 27, 2021.

	Overall		Fully Vaccinated		Not/Partially Vaccinated		p-value
**Sex**							0.06
Female	43	(21)	18	(16)	25	(27)	
Male	164	(79)	95	(84)	69	(73)	
**Age, at diagnosis**							0.01
<12	8	(4)	0	(0)	8	(9)	
12–15	1	(0)	0	(0)	1	(1)	
16–20	28	(14)	14	(12)	14	(15)	
20–29	115	(56)	64	(57)	51	(54)	
30–39	36	(17)	23	(20)	13	(14)	
40+	19	(9)	12	(11)	7	(7)	
**Branch of service/affiliation**							0.005
US Army	161	(78)	96	(85)	65	(69)	
US Air Force	33	(16)	15	(13)	18	(19)	
Other (AAFES, DoD)	13	(6)	2	(2)	11	(12)	
**Position**							0.24
US service member	169	(82)	96	(85)	73	(78)	
Family member	19	(9)	7	(6)	12	(13)	
US personnel (contractor, civilian)	10	(5)	4	(4)	6	(6)	
Affiliated personnel	9	(4)	6	(5)	3	(3)	
**Symptomatic**							0.43
Yes	153	(74)	86	(76)	67	(71)	
No/not reported	54	(26)	27	(24)	27	(29)	
**Location of residence**							0.63
Installation	125	(60)	68	(60)	57	(61)	
Off installation	33	(16)	16	(14)	17	(18)	
Unknown	49	(24)	29	(26)	20	(21)	
**Symptoms** *							
Cough	78	(51)	38	(44)	40	(60)	0.19
Headache	64	(42)	38	(44)	26	(39)	0.37
Congestion	64	(42)	41	(48)	23	(34)	0.07
Fever	52	(34)	25	(29)	27	(40)	0.34
Body ache	36	(24)	18	(21)	18	(27)	0.47
Loss of taste	36	(23)	21	(24)	15	(22)	0.71
Sore/itchy throat	35	(23)	21	(24)	14	(21)	0.58
Loss of smell	32	(21)	18	(21)	14	(21)	0.84
Chills	23	(15)	14	(16)	9	(13)	0.66

Note: n (%); US–United States, AAFES—Army & Air Force Exchange Service, DoD–Department of Defense

*other symptoms reported by less than 10% were fatigue (9%), runny nose (8%), mucus (5%), shortness of breath or nausea or diarrhoea (4% each), sneezing (3%), eye pain or chest pain or rash or loss of appetite or dizziness (1% each).

Among fully vaccinated cases, most (92, 81%) received Janssen, 18 (16%) received Moderna, and 3 (3%) received Pfizer. A mean 84.4 days (range 15.0–172.0) had elapsed to diagnosis of infection. Cases who were fully vaccinated did not differ significantly from those who were unvaccinated in the proportion who experienced symptoms (vaccinated 76% vs unvaccinated 71%, p = 0.43), the median number of symptoms reported (3.0 each, p = 0.57) or in median diagnostic Ct values of specimens (vaccinated 20.0 vs unvaccinated 19.8, p = 0.82).

**Fig 2:**
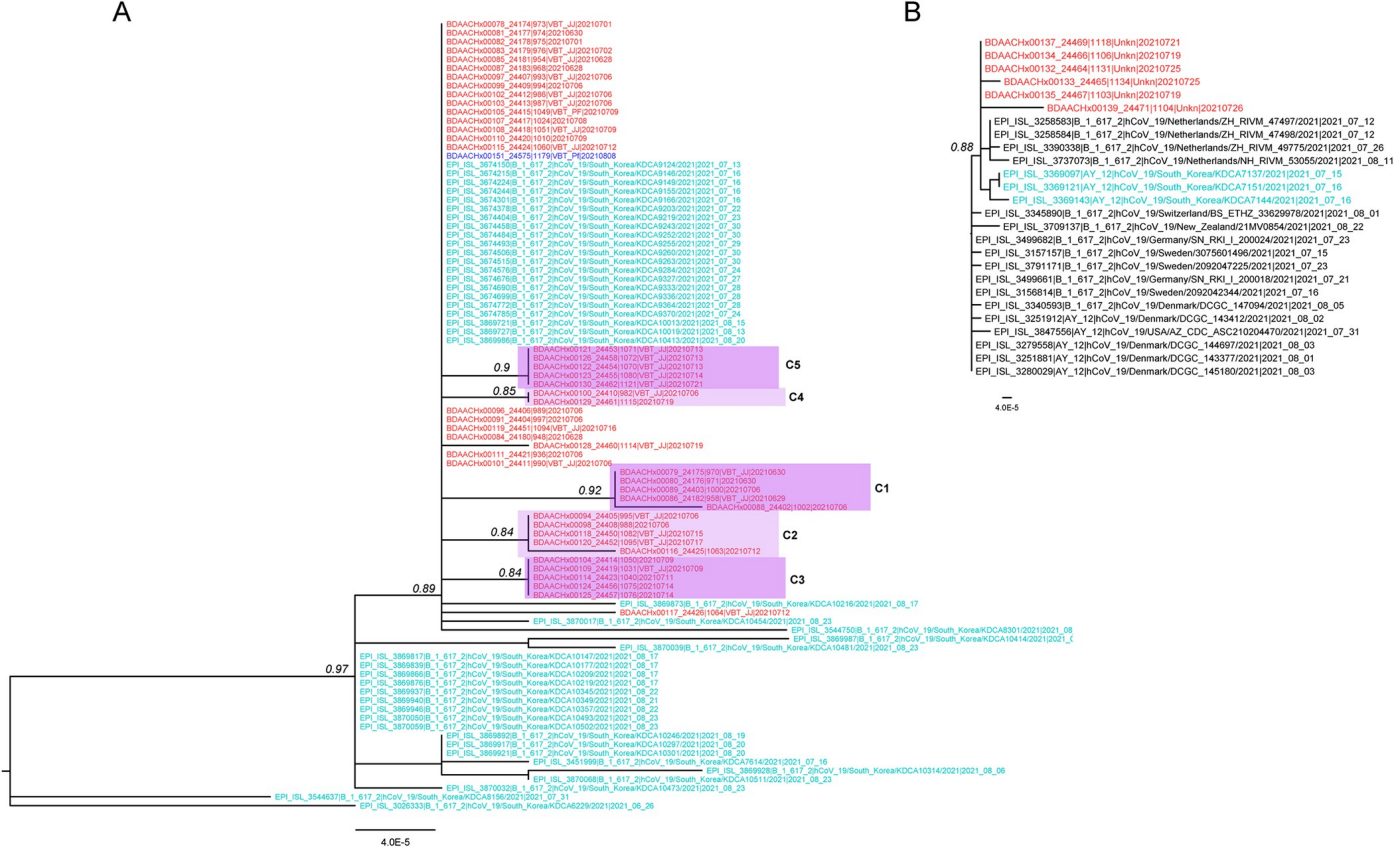
SARS-CoV-2 Delta sub-trees of the two USFK outbreaks. A) Sustained spread involving 86% of the genomes B) Sustained spread involving 9% of the genomes. USFK genomes collected June 27 to July 27 are colored red, those collected July 29 to August 10 are marked in dark blue. Local Korean population genomes are colored teal. Relevant node confidence values are displayed in italic.

**Fig 3:**
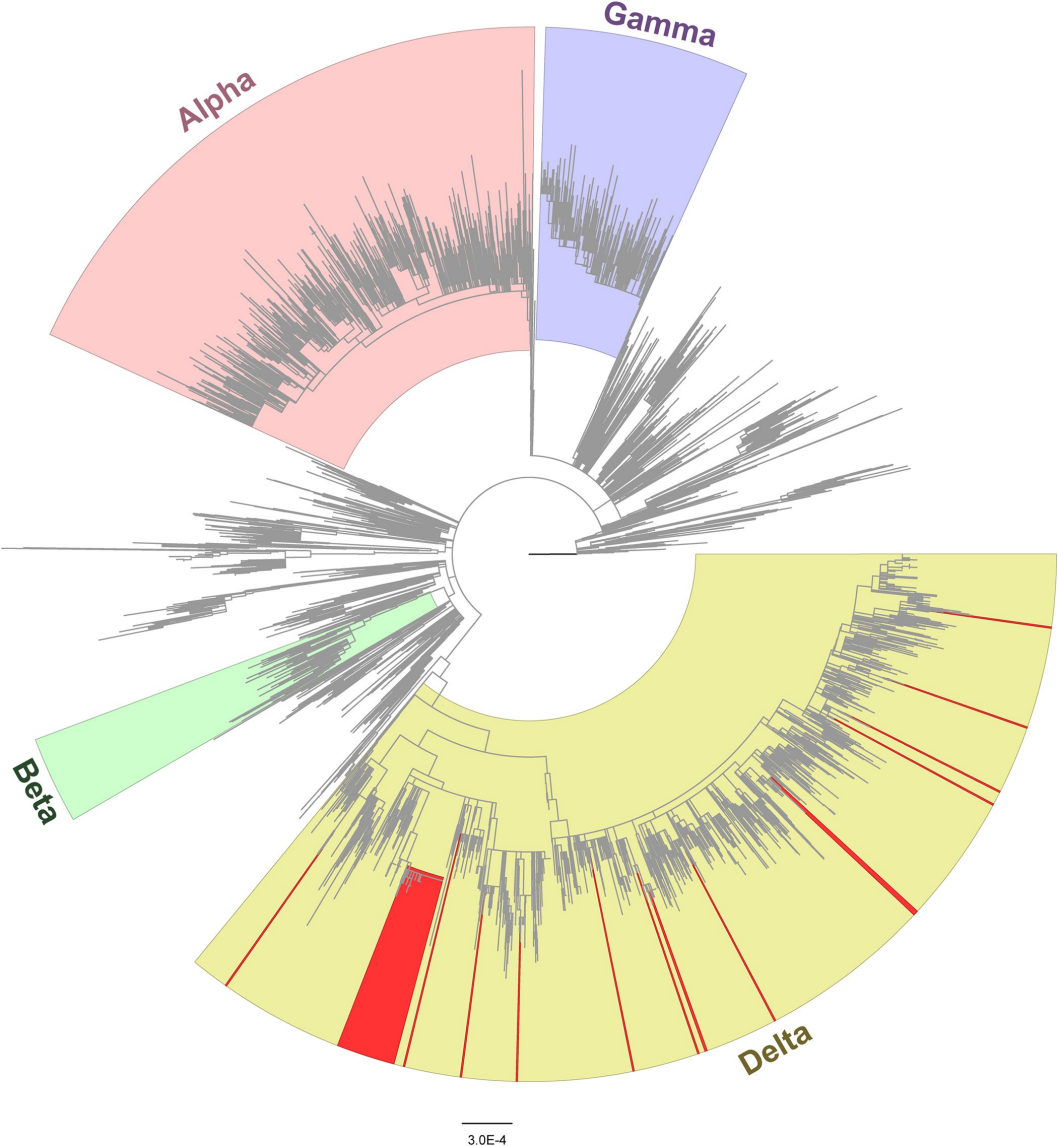
Maximum likelihood phylogenetic tree with highlighted SARS-CoV-2 Alpha, Beta, Gamma and Delta lineages. Genomes from 67 U.S. military personnel and beneficiaries are highlighted in red.

In the time period 27 June to 10 August, 123 NP specimens had Ct values below 31, of which 76 were received for sequencing. A total 67 specimens had sufficient genomic coverage for analysis and 9 could not be sequenced. Of 67 NP specimens, genomic sequencing of 54 collected between 27 June and 27 July indicated all cases were infected with Delta variant (B.1.617.2) as were an additional 13 COVID-19 genomes sampled from 29 July to 10 August. Phylogenetic analysis indicated two introductions led to sustained transmission accounting for 41% (51/123) of infections between 27 June and 27 July. During this time, one introduction resulted in 83% (45/54) infections in the same clade and was closely related to genomes from the local population in the month preceding the outbreak (Fig 2A). Five distinct transmission clusters were observed in this clade among a subset of 22 members, 12 of whom were vaccinated at the time of diagnosis. Another introduction resulted in 11% (6/54) genomes in the same clade, which were also related to local strains; members’ vaccination status was unknown (Fig 2B). The remaining 3 of 54 genomes were not related to local strains. Of the 13 genomes collected July 29–10 August, only one clustered within the large USFK clade, and the remaining 12 genomes represented independent introductions unrelated to local strains and no subsequent sustained spread among USFK (Figs 2A and 3) supporting an end to the outbreak.

## Discussion

The total number of COVID-19 infections diagnosed in the 6-week period of the outbreak described here among USFK/affiliated personnel and beneficiaries at installations in the Republic of Korea represented 56% of all local cases identified in the 1.4 years since the onset of the pandemic. Breakthrough infections accounted for more than half of cases of whom more than three fourths were symptomatic. Vaccinated personnel were not at risk of severe illness; however, the high transmissibility of the Delta variant during the pre-symptomatic phase increased the likelihood of transmission to other vaccinated and unvaccinated members likely accounting for the scale of this outbreak [12]. Of note, no hospitalizations occurred among the predominantly young, military members infected with SARS-CoV-2 and specimens from both vaccinated and unvaccinated members had similar RT-PCR cycling threshold values. Although only 6 individuals had been infected previously, these prior infections were noted equally among unvaccinated, partially and fully vaccinated individuals. The onset of the outbreak was tied to easing of restrictions among vaccinated people leading to an increase in social activities in local Korean establishments where the Delta variant had begun to circulate [13]. To control the spread of COVID-19, USFK implemented enhanced mitigation measures on 9 July mandating indoor mask use at all USFK installations, banning leisure travel to the Seoul metropolitan and other surrounding high transmission areas, and placing bars and clubs off-limits for all personnel regardless of vaccination status. The USFK masking mandate preceded the U.S. Centers for Disease Control and Prevention’s 27 July 2021 recommendation for indoor masking among vaccinated and unvaccinated persons in high COVID-19 transmission locations. The USFK Public Health Emergency declaration was renewed several times, each for a period of 90 days [14]. Multiple public health measures such as rapid separation of infected and exposed members, and enhanced surveillance and testing of affected units were required to control the spread of SARS-CoV-2 before the executive order on 9 September mandating a universal vaccination policy among federal workers could be implemented to achieve 100% coverage over time.

Due to vaccine supply shortages among the ROK general population, only 7% had received at least one dose and only 2% were fully vaccinated at the time of the outbreak [15, 16]. Our findings indicate vaccines were protective against severe illness and hospitalization from infection by the Delta variant. However, concurrent use of nonpharmaceutical interventions such as transmission-reducing behaviors, testing, quarantine, and isolation were needed alongside continued roll out of vaccination programs to prevent the spread of infection. Findings from our investigation parallel those of other outbreaks involving SARS-CoV-2 Delta variant infections among vaccinated individuals [17, 18]. Breakthrough infections comprised 74% of cases and 79% symptomatic infections in a large outbreak in Provincetown, Massachusetts following large summer public events where tourists from geographic locations with varying SARS-CoV-2 transmission mingled with residents in a community where vaccination coverage had reached 69%; Delta variant was identified from 99% of samples sequenced [17, 19]. On 17 July, these data prompted the CDC to recommend mask usage by all persons in indoor public settings in areas with substantial or high transmission [20].

There are strengths and limitations to this report. The strengths of the outbreak response detailed here include swift mobilization of resources and responses by leadership and rapid and extensive infection prevention and control practices. Several measures were executed immediately to control the outbreak, which included case finding by aggressive mass surveillance testing of affected units and comprehensive contact tracing regardless of vaccination status. A risk-based strategy was introduced for handling of contacts based on their vaccination status, time elapsed and distance from a known case. Infection control measures included quarantining of contacts and isolation of laboratory-confirmed cases, implementation of strict cleaning protocols involving sanitizing primary locations visited by confirmed cases such as fitness centers, cafeterias, residential barracks, etc. The USFK leadership quickly re-instated travel bans to Seoul and to bars and clubs among both vaccinated and unvaccinated personnel. Lastly, addition of whole genome sequencing characterized transmission clusters and sources of introduction of the virus. This investigation may have been enhanced by characterizing antibody responses at the time of infection among members who had been vaccinated or had prior COVID-19. Since all USFK/USFK-affiliated members investigated were less than 65 years of age, disease presentation may not be reflective of breakthrough infections among older USFK beneficiaries with comorbidities. Therefore, case counts reported may have been underestimated.

Findings from this investigation suggest transitionary COVID-19 mitigation measures can be successful in controlling spread of the Delta variant and are warranted even as vaccination rates steadily increase among USFK and other U.S. military personnel stationed in the U.S. and world-wide. At the time of the outbreak only 2–7% of the ROK general population had been vaccinated in comparison to 70% of USFK. Vaccine scarcity was not unique to the ROK. Despite the historically unprecedented development and approval of not one but several vaccines within a year of the identification of a new pathogen, administration of 75% of the vaccines were limited to only 10 countries in the month preceding onset of the outbreak [21]. The impact at a global level can be evidenced in the microcosm reflected herein with this account [22]. The effect of unequal vaccine allocation has realized surges in cases as evidenced by the USFK outbreak, diversion of resources from other necessary public health programs to address such emergencies with an inevitable ensuing toll on health, welfare, and morale of the population, and last and not least an increased burden of SARS-CoV-2 in the USFK population from ongoing risk through exposure to communities with continual transmission. Active surveillance and rapid public health response and investigation remain critical to mitigation efforts. Furthermore, as evidenced by this report, other nonpharmaceutical interventions are needed concurrently with vaccination to contain the spread of the virus and to prevent future surges in cases.
